# Effects of dendrobium nobile polysaccharide on reproductive performance, hormone levels, antioxidant capacity, and testicular metabolism of aged roosters

**DOI:** 10.1016/j.psj.2026.107602

**Published:** 2026-08-11

**Authors:** Jilong Zhang, Depeng Zhao, Yushi Shi, Xia Long, Qisong Tan, Jinlin Yang, Gang Feng, Beida Zhou, Hui Li

**Affiliations:** a Key Laboratory of Animal Genetics, Breeding and Reproduction in the Plateau Mountainous Region, Ministry of Education, Guizhou University, Guiyang, 550025, Guizhou Province, China; b College of Animal Sciences, Guizhou University, Guiyang, 550025, Guizhou Province, China

**Keywords:** Dendrobium nobile polysaccharide, Aged breeding rooster, Sperm quality, Reproductive performance, Oxidative stress

## Abstract

As roosters age, semen quality gradually declines, and exacerbated oxidative stress is an important contributing factor to the deterioration of their reproductive function. Dendrobium nobile Lindl. (**DNL**), a traditional Chinese medicine, exhibits potent antioxidant activity and plays an important role in delaying the decline of reproductive function in aging roosters; however, whether its core active component, Dendrobium nobile polysaccharide (**DNP**), serves as the key effective constituent remains to be verified. In this study, twenty 56-week-old male recessive white-feathered chickens (a recessive white-feathered line developed by Guizhou University) were randomly allocated to a control group (**CON**) and a DNP-supplemented group (**DNP**), with 10 birds per group, for a 60d feeding trial to investigate the effects of DNP on antioxidant capacity, hormone levels, testicular metabolism, and reproductive performance of aged roosters. The results demonstrated that, compared with the CON group, DNP supplementation improved semen quality, enhanced antioxidant capacity and serum hormone levels, and delayed testicular tissue degeneration in aged roosters. Untargeted metabolomics analysis suggested that alterations in the testicular metabolic profile of the DNP group were potentially associated with improvements in antioxidant capacity, with differential metabolites primarily enriched in amino acid biosynthesis, aminoacyl-tRNA biosynthesis, and cysteine and methionine metabolism pathways. These findings indicate that dietary DNP supplementation can effectively alleviate testicular oxidative damage and slow the decline in reproductive performance in roosters, highlighting the potential of DNP as a feed additive to enhance antioxidant capacity in breeding roosters.

## Introduction

Prolonging the productive lifespan of breeding roosters yields considerable economic benefits in poultry production. Roosters typically reach peak reproductive performance between 24 and 28 weeks of age. However, as the birds continue to age, a significant challenge confronting roosters is the decline in reproductive performance and semen quality (Hong, 2020). This trend runs counter to the production efficiency pursued by the poultry industry. These declines are attributable, in part, to aging-related reproductive dysfunction, characterized by testicular atrophy, reduced semen volume, decreased sperm motility and concentration, and increased abnormal sperm morphology (Lagares et al., 2017). Studies have shown that with advancing age, reactive oxygen species (**ROS**) production increases in the testes and semen of roosters, while antioxidant defense capacity declines, leading to elevated oxidative stress levels (Liu et al., 2023). Oxidative stress can cause testicular damage, potentially disrupting DNA, RNA, and protein functions in sperm cells and testicular somatic cells, resulting in sperm defects and subsequent damage to spermatozoa (Wang et al., 2025). Sperm cell impairment directly affects semen quality, which, as a key indicator of reproductive performance in breeding roosters, directly undermines fertilization capacity and subsequently influences hatchability and chick quality (Authaida et al., 2023). Therefore, enhancing antioxidant capacity during the rearing period of breeding roosters is a critical strategy for delaying reproductive senescence and maintaining their breeding value.

Dendrobium nobile Lindl. (**DNL**), a traditional Chinese medicinal herb, is rich in bioactive components including alkaloids, phenanthrenes, fluorenones, and polysaccharides, and exhibits favorable biological activities in antioxidant, anti-aging, and anti-inflammatory capacities (Ling et al., 2021). Our research group previously conducted a feeding trial to investigate whether DNL could improve the reproductive performance of breeding roosters, and the results demonstrated that dietary DNL supplementation improved production performance by alleviating testicular oxidative damage (Zhao et al., 2025). Dendrobium nobile polysaccharide (DNP), one of the principal active components of DNL, is present at concentrations far exceeding those of total polyphenols, total flavonoids, and total alkaloids (Li et al., 2022). Previous studies have shown that Dendrobium officinale polysaccharide can mitigate cisplatin-induced oxidative damage to the male reproductive system in mice by upregulating Nrf2 pathway genes (Lu et al., 2019).

However, it remains unclear whether dietary supplementation with DNP can enhance antioxidant capacity and alleviate age-related reproductive decline in breeding roosters. Therefore, building upon our group's previous research, the present study further investigated the effects of DNP on the reproductive performance and testicular antioxidant parameters of breeding roosters, and explored the regulatory mechanisms by which DNP improves reproductive performance through testicular metabolomics analysis. This study aims to facilitate the development and utilization of novel feed additives while providing new approaches for advancing animal welfare and sustainable livestock production.

## Materials and methods

### Dendrobium nobile Polysaccharide (DNP)

The Dendrobium nobile polysaccharide (DNP) used in this experiment was purchased from Yolanda Biological Technology Co., Ltd. (Xi'an, Shaanxi, China), with a detected purity of 80.05%. The remaining 19.95% consisted primarily of bound protein (4.76%), moisture (7.59%), ash (5.00%), and small amounts of total flavonoids (0.28%), total alkaloids (0.28%), total polyphenols (0.48%), free amino acids (0.65%), and residual pigments and other small molecular substances (0.91%).

### Experimental design and diets

Twenty 56-week-old male recessive white-feathered chickens, all in the stage of reproductive decline (Lagares et al., 2017), were selected for this study. The roosters were randomly assigned to 2 groups of 10 birds each: a control group (CON) and a Dendrobium nobile polysaccharide group (DNP). Roosters in the CON group were fed a basal diet, while those in the DNP group received the basal diet supplemented with 1,500 mg/kg DNP. The DNP supplementation level was selected based on our research group's previous studies (Shi et al., 2025; Tan et al., 2024; Yang et al., 2025). The diet formulation met the recommended standards of the Chinese Feed Composition and Nutritional Value Table (35th edition, 2024). The ingredient composition of the basal diet is presented in Table 1. All roosters were obtained from the same batch within the same poultry house and were individually housed in cages with ad libitum access to water and powdered feed. All birds were maintained in a controlled environment at approximately 21°C, 50% to 65% relative humidity, and a photoperiod of 16 h of light per day.

**Table 1:** Ingredients composition and nutrient levels of the basal diet (air-dry basis).

Ingredients	Content, g/kg	Nutrient levels^2^	Content
Corn	740.0	Metabolizable energy, Mcal/kg	2.97
Corn germ meal	50.0	Crude protein (%)	14.06
Soybean meal, 46% CP	120.0	Crude fat (%)	4.12
Soybean oil	10.0	Calcium (%)	0.72
Wheat bran	50.0	Sodium (%)	0.12
Limestone	13.2	Non-phytate phosphorus(%)	0.30
CaHPO4	5.0	Digestible Lysine (%)	0.70
Salt	2.0	Digestible Sulfur Amino Acids (%)	0.52
Sodium bicarbonate	1.0	Digestible Threonine (%)	0.41
Choline chloride (50%)	0.5		
Calcium formate	1.5		
Montmorillonite	1.0		
L-Lysine·HCl (78% Lys)	2.5		
DL-Methionine (99% Met)	0.8		
Bacillus subtilis preparation	0.5		
Phytase (5,000 FTU/kg)	0.2		
Premix^1^	1.8		
**Total**	**1,000.0**		

1 The premix provided the following per kg of the diet: vitamin A, 12,500 IU; vitamin D_3_, 4,000 IU; vitamin E, 30 IU; vitamin K_3_, 2.0 mg; vitamin B_1_, 2.0 mg; vitamin B_2_, 6.0 mg; vitamin B_6_, 3.0 mg; vitamin B_12_, 0.02 mg; niacin, 30 mg; pantothenic acid, 12 mg; folic acid, 1.0 mg; biotin, 0.15 mg; choline chloride, 500 mg; Iron, 80 mg; Copper, 8 mg; Zinc, 60 mg; Manganese, 80 mg; Iodine, 0.35 mg; Selenium, 0.15 mg.

2 Metabolizable energy (ME) and digestible amino acid values in the nutrient levels were calculated values, while the others were measured values. ME values were drawn from the Chinese Feed Composition and Nutritional Value Table (35th edition, 2024), Chinese feed database.

### Semen sample collection

Ten roosters from each group were randomly selected for semen collection by the dorsoventral massage method on days 0, 20, 45, and 60 of the experiment (Guo et al., 2025), and the ejaculate volume of each bird was recorded. A 10 µL aliquot of fresh semen was diluted at 1:200 (v/v) with a sperm diluent containing 3% NaCl (pre-warmed to 37°C) to immobilize spermatozoa for counting. The diluted semen was thoroughly mixed, and 10 µL was loaded into the counting chamber of an improved Neubauer hemocytometer that had been pre-covered with a coverslip (Brillard and McDaniel, 1985). After allowing 3 min for spermatozoa to settle, spermatozoa were counted under a light microscope at 400 × magnification in 5 medium squares (4 corner squares and 1 central square) of the central large square. Two independent diluted samples were prepared for each specimen and counted separately, and the average value was used. Sperm concentration was calculated using the following formula:

$$\text{Sperm}\text{concentration}\left(\text{sperm}/mL\right)=N_{\text{counted}\;}\times D\times 5\times 10^{4}$$

where $N_{\text{counted}}$ represents the total number of spermatozoa counted in the five middle squares, and D denotes the dilution factor. The coefficient $5\times 10^{4}$ converts the counted volume of five medium squares (0.02 mm³) to 1 mL.

Sperm motility was evaluated using a microscopic subjective scoring method (Shayan-Nasr et al., 2021). Within 10 min after semen collection, samples were temporarily stored in a 37°C water bath and transported to the laboratory. A 10 µL aliquot of fresh semen was diluted at 1:10 (v/v) with physiological saline pre-warmed to 37°C; 10 µL of the diluted semen was placed on a pre-warmed glass slide, gently covered with a coverslip, and observed under a light microscope equipped with a thermostatic stage at 400 × magnification. Five random fields were observed per sample, and the percentage of spermatozoa exhibiting progressive forward motion relative to the total number of spermatozoa in the field was assessed. Each sample was measured in duplicate, and the average value was used. All assessments were completed within 30 min after semen collection (Mortimer and Mortimer, 2012). Sperm motility was expressed as the percentage of progressively motile spermatozoa (%). Effective sperm concentration was calculated from sperm concentration and sperm motility using the following formulas:

$$\text{Sperm}\;\text{motility}\;(\%)=\frac{N_{\text{progressive}}}{N_{\text{total}}}\times \;100$$

where $N_{\text{progressive}}$ is the number of progressively motile spermatozoa, and $N_{\text{total}}$ is the total number of spermatozoa counted.

$$\text{Effective}\;\text{sperm}\;\text{concentratio}n_{\text{sperm}/\text{mL}}=C_{\text{sperm}}\times \frac{M_{\text{sperm}}}{100}$$

where $C_{\text{sperm}}$ represents the sperm concentration (sperm/mL), and $M_{\text{sperm}}$ denotes the sperm motility (%).

### Hatchability determination

On 45 d of the experiment, semen was collected from the breeding roosters at a frequency of every other day. After two consecutive collections, semen from roosters within the same group was pooled in equal volumes to prepare mixed semen. The mixed semen was temporarily stored in a 37°C water bath, and artificial insemination was completed within 30 min after semen collection (Donoghue and Wishart, 2000; Sexton, 1987). Hens reared under identical conditions and from the same batch were selected, and artificial insemination was performed via the intravaginal route at 14:00 (Watson, 1998), with an insemination dose of 0.025 mL per hen and an insertion depth of approximately 2 cm. Each hen was inseminated twice consecutively at 5 d intervals. Egg collection began on the third day after the first insemination, with eggs collected twice daily. A total of 150 eggs meeting hatchery standards were selected from each group for incubation, divided into 3 batches of 50 eggs each. Incubation was conducted at 37.5°C with 55% to 65% relative humidity, and eggs were automatically turned once per hour. On 7 d of incubation, eggs were candled to record the numbers of unfertilized eggs, dead embryo eggs, and fertile eggs for calculating the fertility rate. On 18 d, eggs were transferred to the hatcher and dead embryo eggs were removed. On 21 d, the number of hatched chicks was recorded to calculate the hatchability of fertile eggs and hatchability of settable eggs. The formulas are as follows:

$$\text{Fertility}\;\text{rate}\;(\%)=\frac{N_{\text{fertile}}}{N_{\text{set}}}\times \;100$$

$$\text{Hatchability}\;\text{of}\;\text{fertile}\;\text{eggs}\;(\text{HFE},\;\%)=\frac{N_{\text{hatched}}}{N_{\text{fertile}}}\times \;100$$

$$\text{Hatchability}\;\text{of}\;\text{settable}\;\text{eggs}\;(\text{HSE},\;\%)=\frac{N_{\text{hatched}}}{N_{\text{set}}}\times \;100$$

where $N_{\text{set}}$ is the number of settable eggs placed in the incubator; $N_{\text{fertile}}$ is the number of fertile eggs identified by candling on 7 d of incubation; and $N_{\text{hatched}}$ is the number of chicks hatched from those eggs.

### Blood sample collection

Blood samples were collected from the experimental animals via the wing vein on days 0, 40, and 60 of the experiment. All 20 roosters were fasted the day before blood collection. A 4 mL blood sample was collected from each bird and centrifuged at 3,000 r/min for 15 min at 4°C. The separated serum samples were stored at −80°C. Subsequently, all 20 roosters were weighed and then humanely euthanized by cervical dislocation. Immediately after euthanasia, the testes were removed. Both testes were weighed using an electronic balance, and the testis index was calculated (Mfoundou et al., 2022). A portion of the testicular tissue was fixed in 4% paraformaldehyde solution for histological analysis, while the remaining tissue was stored at −80°C for gene expression analysis, antioxidant parameter measurement, and metabolomics analysis. The testis index was calculated using the following formula:

$$\text{Testis}\;\text{index}\;(\%)=\frac{W_{\text{testes}}}{W_{\text{body}}}\times \;100$$

$W_{\text{testes}}$ = weight of both testes (g);$W_{\text{body}}$ = live body weight of the rooster (g).

### Reproductive hormone assay

Serum concentrations of follicle-stimulating hormone (**FSH**), luteinizing hormone (**LH**), and testosterone were determined using enzyme-linked immunosorbent assay (ELISA) kits (Nanjing Jiancheng Bioengineering Institute, Nanjing, China). Absorbance was measured using a Synergy H4 microplate reader (BioTek Instruments, Winooski, VT).

### Antioxidant parameter measurement

Commercial kits (Nanjing Jiancheng Bioengineering Institute, Nanjing, China) were used to measure the activities of glutathione peroxidase (**GSH-Px**), total superoxide dismutase (**T-SOD**), catalase (**CAT**), and total antioxidant capacity (**T-AOC**) in serum samples. The level of malondialdehyde (**MDA**), a lipid peroxidation product, was analyzed by spectrophotometry. For testicular antioxidant analysis, 0.2 g of frozen testicular tissue was ground and homogenized in 2 mL of physiological saline. The homogenate was then centrifuged at 12,000 × g for 10 min at 4°C, and the supernatant was collected for antioxidant measurement.

### Real-time quantitative PCR (RT-qPCR) analysis

Total RNA was extracted from 0.1 g of testicular tissue using TRIzol reagent (Invitrogen, Carlsbad, CA) according to the manufacturer's protocol. The purity and integrity of the isolated RNA were assessed by agarose gel electrophoresis, and the total RNA concentration was determined using a spectrophotometer. For reverse transcription, single-stranded cDNA was synthesized using a First-Strand cDNA Synthesis Kit (Cat# K1621; Thermo Fisher Scientific, Waltham, MA). The relative expression levels of Nrf2 pathway-related genes—including nuclear factor erythroid 2-related factor 2 (**Nrf2**), superoxide dismutase 1 (**SOD1**), catalase (**CAT**), NAD(P)H quinone dehydrogenase 1 (**NQO-1**), heme oxygenase 1 (**HO-1**), glutathione S-transferase alpha 3 (**GSTA3**)—and the testosterone synthesis-related gene 17β-hydroxysteroid dehydrogenase 3 (**HSD17B3**) in testicular tissue were determined using a StepOnePlus Real-Time PCR System (Applied Biosystems, Foster City, CA). The qRT-PCR thermal cycling conditions were as follows: pre-denaturation at 95°C for 2 min; 40 cycles of denaturation at 95°C for 15 s, annealing at 60°C for 15 s, and extension at 72°C for 1 min; and a final extension at 72°C for 5 min. The primer sequences used in this study are listed in Supplementary Table 1. The relative expression levels of target genes were determined using the 2^−ΔΔCt^ method, normalized to the mRNA level of the reference gene *β-actin* (Shen et al., 2026).

### Testicular histomorphology

Histological analysis of rooster testes was performed using hematoxylin and eosin (**H&E**) staining. The collected left testis was cut into small blocks (1.0 cm × 1.0 cm × 0.5 cm) and fixed in 4% paraformaldehyde solution for 48 h. The samples were then embedded in paraffin blocks, sectioned at 5 µm thickness, and stained with H&E (Wang et al., 2025). Two sections were prepared per testicular sample, and 28 seminiferous tubules with regular contours were randomly selected. Seminiferous tubule diameters were measured at 400 × magnification using CaseViewer 2.3 (Servicebio Technology Co., Ltd., Wuhan, Hubei, China).

### Testicular metabolomic analysis

Testicular metabolites were analyzed using an untargeted metabolomics approach. After thawing samples slowly at 4°C, a portion of each sample was homogenized with an appropriate volume of methanol/acetonitrile/water solution (2:2:1, v/v/v). The mixture was subjected to low-temperature ultrasonication for 30 min, incubated at −20°C for 10 min, and then centrifuged at 14,000 × g for 20 min at 4°C. The supernatant was vacuum-dried and reconstituted in 100 µL of acetonitrile-water solution (1:1, v/v). After vortexing and a second centrifugation for 15 min, the supernatant was collected for subsequent analysis. LC-MS analysis was performed using an Agilent 1290 Infinity UHPLC system coupled with an AB TripleTOF 6600 mass spectrometer, with data acquisition in both ESI positive and negative ion modes. Raw mass spectrometry data were processed for peak extraction, alignment, and normalization to construct a data matrix. Principal component analysis (**PCA**) and orthogonal partial least squares discriminant analysis (**OPLS-DA**) were used to evaluate group separation trends, and variable importance in projection (**VIP**) values were extracted from the OPLS-DA model. Differential metabolites were screened using the thresholds of VIP > 1.0, |FC| > 1.5, and P < 0.05, yielding a total of 55 differential metabolites. KEGG pathway enrichment analysis was performed on the differential metabolites, and Spearman correlation analysis was used to assess the associations between differential metabolites and semen quality parameters, antioxidant indices, and antioxidant gene expression to elucidate the mechanisms of testicular metabolic perturbation.

### Statistical analysis

Routine statistical analyses were performed using IBM SPSS Statistics 25.0 software. All continuous variables were tested for normality using the Shapiro-Wilk test. Data following a normal distribution were analyzed using the independent-samples t-test, while non-normally distributed data were analyzed using the Mann-Whitney U test. Categorical variables were compared using the chi-square test. For group comparisons of the same variable across multiple time points, P-values were adjusted using the Benjamini-Hochberg false discovery rate (**FDR**) correction to control for multiple comparison errors. Spearman correlation coefficients were used to analyze the correlations among semen quality, testicular antioxidant indices, antioxidant gene mRNA expression, and differential metabolites. For the untargeted testicular metabolomics data, OPLS-DA was performed using the MetaboAnalystR package in R 4.1.2 to extract VIP values; the screening criteria for differential metabolites were VIP > 1.0, |FC| > 1.5, and P < 0.05. All data are expressed as mean ± standard error of the mean (**SEM**). The level of significance was set at P < 0.05. Metabolomics differential analysis and visualization were performed using an online tool (https://www.omicstudio.cn/tool).

## Results

### Semen quality parameters

As shown in Table 2, on 0 d of the experiment, there were no differences between the CON and DNP groups in semen volume, sperm concentration, sperm motility, or effective sperm concentration. As the experimental period progressed, the sperm concentration, sperm motility, and effective sperm concentration of CON group roosters showed a declining trend. By 45 d, the sperm concentration, sperm motility, and effective sperm concentration of the DNP group were all higher than those of the CON group (P = 0.014, P = 0.016, and P < 0.001). By 60 d, the DNP group still showed higher sperm motility (P = 0.029), effective sperm concentration (P < 0.001), and semen volume (P = 0.031) compared with the CON group, whereas sperm concentration did not differ between the two groups (P = 0.061). Throughout the experiment, semen volume did not differ between the two groups on days 0, 20, and 45 (P = 0.672, 0.212, and 0.135).

**Table 2:** Effect of dietary DNP supplementation on semen quality parameters of roosters.

Item	Day	CON	DNP	SEM	P-value
Semen volume (mL)	0	0.341	0.357	0.026	0.672
	20	0.305	0.351	0.025	0.212
	45	0.293	0.349	0.025	0.135
	60	0.283	0.369	0.026	0.031
Sperm concentration (× 10⁹/mL)	0	2.18	1.93	0.214	0.423
	20	2.37	2.44	0.278	0.870
	45	2.58	3.45	0.228	0.014
	60	2.45	3.34	0.314	0.061
Sperm motility(% of forward progressive sperm)	0	50.4	47.8	2.82	0.527
	20	47.5	50.9	2.72	0.398
	45	43.3	52.3	2.38	0.016
	60	43.2	51.8	2.57	0.029
Effective sperm concentration (× 10⁹/mL)	0	1.09	0.952	0.136	0.473
	20	1.11	1.25	0.162	0.970
	45	1.12	1.78	0.119	<0.001
	60	0.982	1.74	0.135	<0.001

1 CON = basal diet; DNP = basal diet supplemented with Dendrobium nobile Polysaccharide.

2 SEM = standard error of the mean (n = 10 roosters per treatment per time point).

P-values were obtained from independent samples t-test between CON and DNP at each time point.

### Fertility, hatchability, and serum hormone levels

As shown in Table 3, the fertility rate, hatchability of settable eggs, and hatchability of fertile eggs of CON group roosters were 80.0%, 71.3%, and 89.2%, respectively, while those of the DNP group were 84.7%, 80.7%, and 95.3%, respectively. The fertility rate of the DNP group was 4.7 percentage points higher than that of the CON group (P = 0.364), the hatchability of settable eggs was 9.4 percentage points higher (P = 0.079), and the hatchability of fertile eggs was 6.1 percentage points higher (P = 0.118); however, none of these three parameters reached statistical significance between the groups. The serum hormone levels measured on 60 d of the experiment are presented in Table 3. The serum testosterone level of DNP group roosters was higher than that of the CON group (P = 0.024), and the follicle-stimulating hormone (FSH) level was also higher than that of the CON group (P < 0.001). There was no difference in luteinizing hormone (LH) levels between the two groups (P = 0.503).

**Table 3:** Effect of dietary DNP supplementation on reproductive performance and serum hormone levels of roosters.

Item	CON	DNP	SEM	P-value
**Reproductive traits**				
Fertility rate (%)	80.0	84.7	4.40	0.364
Hatchability of settable eggs (%)	71.3	80.7	4.90	0.079
Hatchability of fertile eggs (%)	89.2	95.3	3.41	0.118
**Serum hormone levels**				
FSH (ng/mL)	49.9	60.6	1.57	<0.001
LH (ng/mL)	3.94	3.72	0.222	0.503
T (ng/mL)	22.4	29.9	2.11	0.024

1 CON = basal diet; DNP = basal diet supplemented with Dendrobium nobile Polysaccharide.

2 SEM = standard error of the mean (n = 150 eggs per group for reproductive traits; n = 8 roosters per group for serum hormone levels).

P-values for reproductive traits were obtained from chi-square test; P-values for serum hormone levels were obtained from independent samples t-test between CON and DNP groups.

### Testicular histomorphology

**Figure 1:**
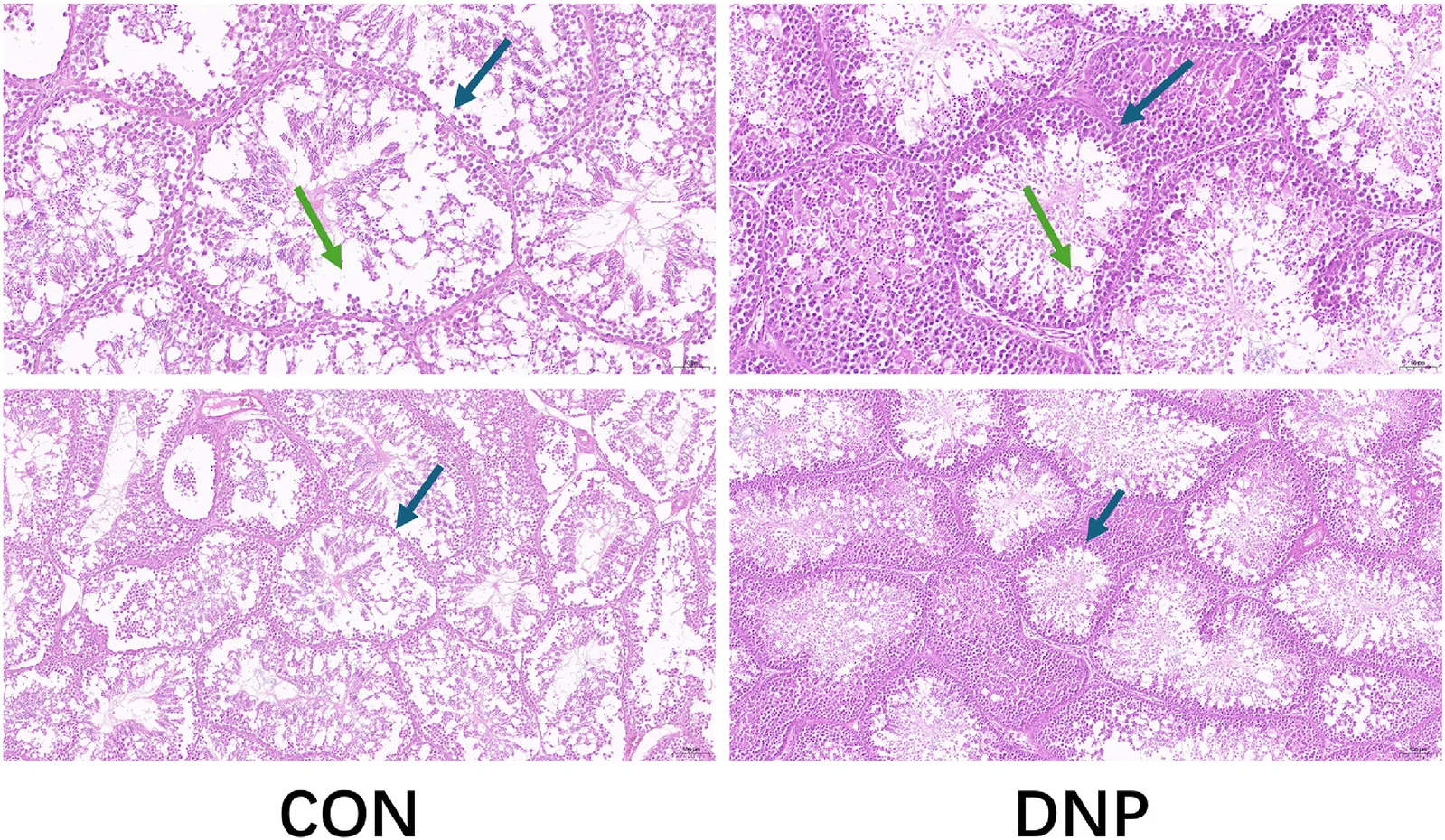
Representative H&E-stained testicular sections from CON (left panels) and DNP (right panels) groups. Upper panels: low-magnification overview of seminiferous tubule architecture (scale bar = 100 μm). Lower panels: high-magnification view of the seminiferous epithelium (scale bar = 50 μm). Green arrows indicate the lumen of seminiferous tubules; blue arrows indicate the seminiferous epithelium.

As shown in Figure 1, H&E staining revealed that the testes of CON group roosters exhibited age-related degenerative changes, including irregular seminiferous tubule contours, thinning of the seminiferous epithelium, loosely arranged spermatogenic cells at various stages, conspicuous seminiferous epithelium (blue arrows), and lumen of seminiferous tubules (green arrows). Compared with the CON group, the testicular morphological features of DNP group roosters were markedly improved, with regularly structured seminiferous tubules and clear boundaries, intact and thickened seminiferous epithelium layers, increased numbers of Sertoli cells and sperm cells, tightly packed cell arrangements, reduced vacuolization, and more compact interstitial tissue. As shown in Table 5, both the testis weight and the mean seminiferous tubule diameter of DNP group roosters were higher than those of the CON group (P = 0.031 and P = 0.012), whereas the testis index did not differ between the two groups (P = 0.065).

### Serum antioxidant enzyme activities

As shown in Table 4, on 0 d, there were no differences between the CON and DNP groups in serum CAT activity, GSH-Px activity, or MDA levels; however, the T-SOD activity of the CON group was higher than that of the DNP group (P < 0.001). On 40 d, the CAT activity of the DNP group was higher than that of the CON group (P < 0.001), GSH-Px activity tended to be higher than that of the CON group (P = 0.062), and serum MDA levels were lower than those of the CON group (P < 0.001); meanwhile, the T-SOD activity of the CON group remained higher than that of the DNP group (P = 0.008). By 60d, both CAT activity (P < 0.001) and GSH-Px activity (P = 0.002) of the DNP group were higher than those of the CON group, MDA levels were lower than those of the CON group (P < 0.001), and there was no difference in T-SOD activity between the two groups.

**Table 4:** Effect of dietary DNP supplementation on serum antioxidant parameters of roosters.

Item	Day	CON	DNP	SEM	P-value
CAT(U/mg prot)	0	2.12	2.13	0.123	0.955
	40	2.08	5.86	0.233	<0.001
	60	1.82	6.83	0.149	<0.001
GSH-Px(U/L)	0	909	906	5.90	0.738
	40	907	916	2.96	0.062
	60	903	933	1.18	0.002
MDA(nmol/mL)	0	8.24	8.52	0.340	0.568
	40	7.88	3.52	0.508	<0.001
	60	9.05	1.69	0.516	<0.001
T-SOD(U/mL)	0	143	129	1.26	<0.001
	40	140	132	1.73	0.008
	60	139	137	1.43	0.745

1 CON = basal diet; DNP = basal diet supplemented with Dendrobium nobile Polysaccharide.

2 SEM = standard error of the mean (n = 8 roosters per treatment per time point).

P-values were obtained from independent samples t-test or Mann-Whitney U test between CON and DNP groups at each time point, with P-values adjusted by the Benjamini-Hochberg false discovery rate (FDR) correction.

### Testicular antioxidant indices and mRNA relative expression

As shown in Table 5, the activities of CAT, GSH-Px, T-SOD, and the level of T-AOC in the testicular tissue of the DNP group were all higher than those of the CON group (P < 0.001, P = 0.008, P < 0.001, and P = 0.010), whereas the MDA level was lower than that of the CON group (P < 0.001). Regarding the mRNA expression of antioxidant-related genes, the relative mRNA expression levels of Nrf2 (P < 0.001), NQO-1 (P = 0.004), GSTA3 (P = 0.007), SOD1 (P < 0.001), and CAT (P = 0.002) in the DNP group were all higher than those in the CON group, while HO-1 expression did not differ between the two groups. Additionally, the relative mRNA expression level of HSD17B3 in the DNP group was higher than that in the CON group (P = 0.003).

**Table 5:** Effects of DNP on testicular parameters, antioxidant gene relative expression, and testis antioxidant indicators of roosters.

Item	CON	DNP	SEM	P-value
**Testicular parameters**				
Testis weight (g)	0.767	1.00	0.067	0.031
Testis index (%)	20.2	24.8	1.65	0.065
Seminiferous tubule diameter (μm)	280	297	5.08	0.012
**Antioxidant gene relative expression**				
Nrf2	1.01	4.34	0.259	<0.001
NQO-1	1.07	3.59	0.482	0.004
HO-1	1.01	1.72	0.340	0.170
GSTA3	1.02	2.80	0.369	0.007
SOD1	1.03	4.75	0.478	<0.001
CAT	1.06	3.54	0.374	0.002
HSD17B3	1.02	3.24	0.401	0.003
**Testis antioxidant indicators**				
CAT(U/mg prot)	1.12	2.40	0.160	<0.001
GSH-Px(U/L)	74.1	82.3	1.94	0.008
MDA(nmol/mL)	1.04	0.572	0.080	<0.001
T-AOC(U/mL)	1.73	1.94	0.052	0.010
T-SOD(U/mL)	18.7	19.6	0.112	<0.001

1 CON = basal diet; DNP = basal diet supplemented with Dendrobium nobile Polysaccharide.

2 SEM = standard error of the mean (n = 10 per group for testis weight and testis index; n = 28 cross-sections per group for seminiferous tubule diameter; n = 6 per group for gene relative expression; n = 10 per group for testis antioxidant indicators).

P-values were obtained from independent-samples t-test or Mann-Whitney U test between CON and DNP groups based on normality.

### Correlation analysis between semen quality and antioxidant parameters

**Figure 2:**
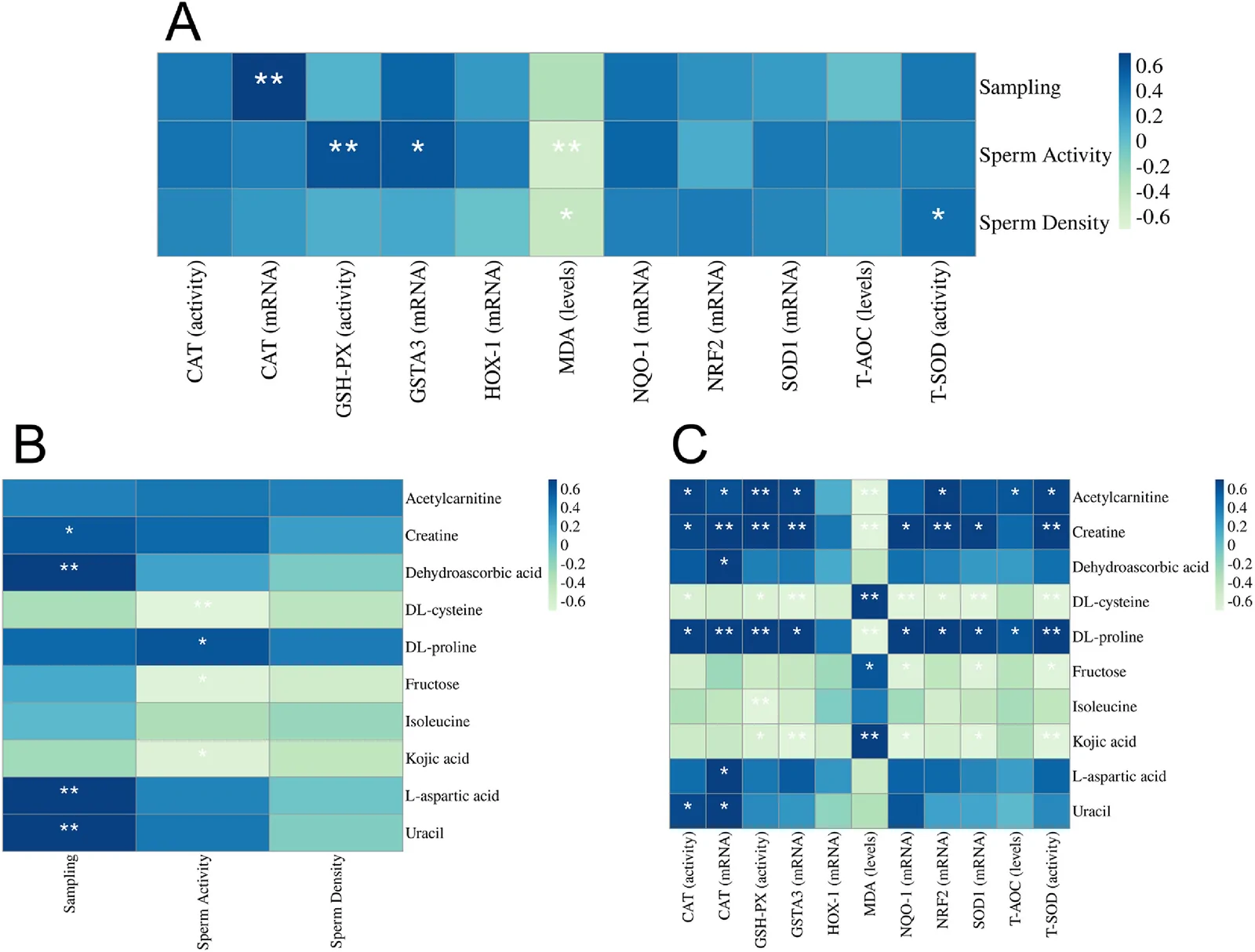
Spearman’s correlation analysis heatmap. Panel (A) shows the correlation heatmap between semen quality parameters and testicular antioxidant indices. The heatmap displays the correlation coefficients among variables, with the color gradient from green to blue indicating increasing correlation coefficients. Panel (B) shows the correlation heatmap between key differential metabolites and semen quality parameters. Panel (C) shows the correlation heatmap between key differential metabolites and testicular antioxidant indices. Statistical significance (*p < 0.05, **p < 0.01, ***p < 0.001).

As shown in Figure 2A, Spearman correlation analysis revealed correlations between testicular antioxidant indices and semen quality parameters. MDA levels were negatively correlated with both sperm motility (P < 0.01) and sperm concentration (P < 0.05), indicating that increased lipid peroxidation is closely associated with reduced sperm motility and concentration. Conversely, CAT mRNA expression levels were positively correlated with semen volume (P < 0.01), and GSH-Px activity was positively correlated with both sperm motility (P < 0.01) and sperm concentration (P < 0.05). Furthermore, GSTA3 mRNA expression levels (P < 0.05) and T-SOD activity (P < 0.05) were both positively correlated with sperm motility, and T-SOD activity was also positively correlated with sperm concentration (P < 0.05).

### Untargeted metabolomics analysis of testicular tissue

**Figure 3:**
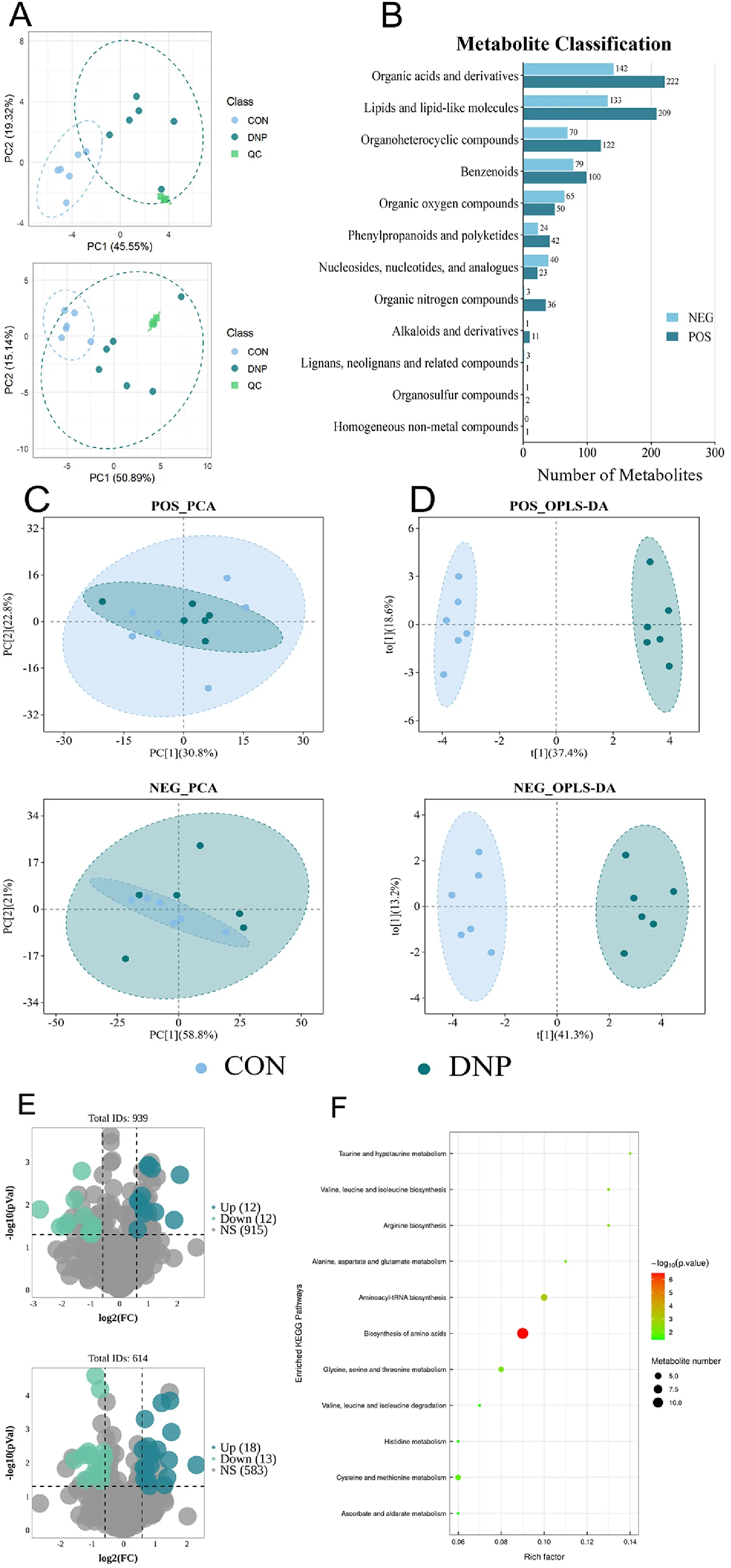
Effects of DNP treatment on testicular tissue metabolism revealed by untargeted metabolomics. Panel (A) Overview PCA score plots showing the overall clustering patterns in positive ion (POS) and negative ion (NEG) modes, including the clustering of quality control (QC) samples. Panel (B) Chemical classification statistics of identified metabolites. The numbers of metabolites in each chemical category in POS and NEG modes are shown. Panel (C) PCA score plots in POS and NEG modes. Panel (D) OPLS-DA score plots in POS and NEG modes. Panel (E) Volcano plots showing the screening results of differential metabolites. Up, up-regulated metabolites; Down, down-regulated metabolites; NS, non-significant metabolites. Panel (F) KEGG pathway enrichment analysis bubble plots. The x-axis represents the rich factor, and the y-axis represents the enriched metabolic pathways; the bubble size represents the number of differential metabolites contained in each pathway, and the color gradient from green to red indicates increasing -log₁₀(p value).

Through untargeted metabolomics analysis, a total of 1,555 metabolites were identified in the testes of CON and DNP group roosters. As shown in Figure 3A, in both positive and negative ion modes, the quality control (**QC**) samples exhibited tight clustering, indicating good instrumental analytical stability; meanwhile, the CON and DNP groups showed clear separation in spatial distribution, suggesting differences in metabolic profiles between the two groups. Based on the HMDB database, all identified metabolites were classified by chemical category: the most abundant categories were organic acids and derivatives and lipids and lipid-like molecules, followed by organoheterocyclic compounds, benzenoids, and others (Figure 3B).

To further analyze the metabolic differences between the two groups, multivariate pattern recognition analysis was performed separately on the positive and negative ion mode data. PCA results showed that the CON and DNP groups exhibited a separation trend in spatial distribution, albeit with some overlapping regions (Figure 3C). The OPLS-DA score plots showed that the two groups clustered on opposite sides of the confidence ellipses, with better separation than PCA (Figure 3D). Using the screening criteria of VIP > 1.0, |FC| > 1.5, and P < 0.05, the volcano plots revealed that 31 differential metabolites were identified in the positive ion mode, of which 18 were upregulated and 13 were downregulated; 24 differential metabolites were identified in the negative ion mode, of which 12 were upregulated and 12 were downregulated (Figure 3E). KEGG pathway enrichment analysis showed that the differential metabolites were primarily enriched in amino acid biosynthesis, aminoacyl-tRNA biosynthesis, cysteine and methionine metabolism, and glycine, serine, and threonine metabolism pathways (Figure 3F).

Based on expression differences and biological functions, 10 key metabolites related to reproduction and antioxidant capacity were selected for correlation analysis. As shown in Figure 2B, semen volume was positively correlated with dehydroascorbic acid, L-aspartic acid, and uracil (P < 0.01), and with creatine (P < 0.05). Sperm motility was negatively correlated with DL-cysteine (P < 0.01) and with fructose (P < 0.05); conversely, it was positively correlated with DL-proline (P < 0.05), and DL-proline was also positively correlated with sperm concentration (P < 0.05). As shown in Figure 2C, acetylcarnitine, creatine, and DL-proline were positively correlated with most antioxidant indices, including CAT, GSH-Px, and T-SOD activities, as well as GSTA3, NQO-1, and SOD1 mRNA expression levels (P < 0.01 or P < 0.05), and negatively correlated with MDA levels. Conversely, DL-cysteine, fructose, and kojic acid were primarily negatively correlated with the aforementioned antioxidant indices (P < 0.01 or P < 0.05) and positively correlated with MDA levels.

## Discussion

The results of this study demonstrate that dietary supplementation with Dendrobium nobile polysaccharide (DNP) improved semen quality, testicular histomorphology, serum reproductive hormone levels, and antioxidant capacity in aged roosters. During the aging process, oxidative stress resulting from the accumulation of reactive oxygen species (ROS) is a key driving factor underlying the decline of reproductive performance in breeding roosters (Asghari-Moghadam et al., 2025). Multiple studies have demonstrated that natural antioxidants such as gallic acid, lycopene, and curcumin can improve semen quality and reproductive performance in aged roosters by alleviating oxidative stress (Behboodi et al., 2024; Ghadimi et al., 2024; Long et al., 2025). In the present study, DNP, a polysaccharide component extracted from the traditional Chinese medicinal herb Dendrobium nobile, exhibited a systemic antioxidant improvement pattern similar to that of the aforementioned natural products. Furthermore, the present study observed a coordinated upregulation of Nrf2 pathway-related genes and HSD17B3 expression in the DNP group, and this expression pattern suggests that Nrf2 pathway activation may be associated with HSD17B3-mediated testosterone synthesis. Metabolomics analysis further suggested a potential association between alterations in the testicular metabolic profile and improvements in antioxidant capacity in the DNP group. These findings provide preliminary experimental evidence supporting DNP as a functional feed additive for extending the reproductive lifespan of breeding roosters.

Semen quality is a core indicator for evaluating the reproductive potential of breeding roosters (Pourazadi et al., 2022). In the present study, the DNP group exhibited improvements in sperm concentration, motility, and effective sperm concentration as early as 45 d of the experiment, and by 60 d, sperm motility and effective sperm concentration remained higher, with semen volume also increased. This time-dependent improvement pattern suggests that the positive effects of DNP on semen quality may require a certain accumulation period. Lycopene supplementation in aged roosters has been shown to improve semen quality (Long et al., 2025), and induced molting can also restore testicular function in aged roosters (Zhu et al., 2023), both exhibiting similar time-dependent improvement patterns. Notably, the difference in sperm concentration between the two groups disappeared by 60 d (P = 0.061), which may reflect the persistent influence of the aging process. Although DNP attenuated the declining trend in sperm concentration, it did not fully reverse the spermatogenic decline associated with aging. The improvement in sperm motility is of direct significance for terminal reproductive outcomes, as sperm motility is a key kinetic parameter determining the ability of spermatozoa to traverse the female reproductive tract, penetrate the vitelline membrane, and complete fertilization (Froman et al., 1999).

Although the fertility rate, hatchability of settable eggs, and hatchability of fertile eggs of the DNP group were 4.7, 9.4, and 6.1 percentage points higher than those of the control group, respectively, none of these three parameters reached statistical significance. This result is commonly observed in studies on aged roosters. While phytogenic blend supplements can improve semen quality parameters, the magnitude of improvement in fertility rate and hatchability is similarly limited (Asghari-Moghadam et al., 2025). The phenomenon whereby the magnitude of improvement in semen quality exceeds that of terminal reproductive indicators may be attributable to the greater sensitivity of semen parameters to supplementation, which can produce significant changes over a relatively short period (Barbarestani et al., 2025). Hatchability is influenced not only by paternal sperm quality but also by multiple factors including maternal egg quality and incubation conditions; demonstrating statistical significance in its improvement requires larger sample sizes and longer intervention periods (Long et al., 2024). Nevertheless, the hatchability of fertile eggs in the DNP group showed an improvement compared with the control group, which still holds considerable economic value in practical production.

The improvement in testicular histomorphology in the DNP group may be structurally associated with the maintenance of spermatogenesis. Both testis weight and seminiferous tubule diameter were increased in the DNP group, with intact seminiferous epithelium layers, tightly packed cells, and reduced vacuolization; these changes were mutually corroborated by the elevated serum testosterone and FSH levels. Adequate testosterone not only supports the differentiation and maturation of spermatogenic cells but also provides a favorable microenvironment for spermatogenesis by maintaining Sertoli cell function and blood-testis barrier integrity (Walker, 2011). FSH acts primarily on Sertoli cells, regulating blood-testis barrier formation, nutritional supply to spermatogenic cells, and sperm release processes (Plant and Marshall, 2001). The coordinated elevation of testosterone and FSH in the DNP group constituted a dual hormonal drive supporting spermatogenesis. Notably, these hormonal changes were closely associated with the between-group differences in antioxidant capacity. Oxidative stress is a key mediating factor linking aging to hypothalamic-pituitary-gonadal (**HPG**) axis dysfunction (Avital-Cohen et al., 2013). At the pituitary level, GnRH receptor activation itself generates ROS as signaling molecules involved in the regulation of gonadotropin synthesis; however, gonadotropes require effective antioxidant defense mechanisms to scavenge excess ROS to maintain normal FSH and LH gene expression (Kim et al., 2019). At the molecular level, the mRNA expression of HSD17B3 was upregulated in the testicular tissue of the DNP group. HSD17B3 is the key rate-limiting enzyme catalyzing the conversion of androstenedione to testosterone, localized in testicular Leydig cells, and is crucial for maintaining testosterone synthesis (Rebourcet et al., 2020). This finding provides associative evidence at the molecular level for the elevated serum testosterone levels, consistent with the finding that astaxanthin promotes testosterone secretion by upregulating HSD17B3 expression in Leydig cells of aged roosters (Liu et al., 2026). The activation of the Nrf2 pathway may be associated with this regulatory process. In poultry, lycopene improves antioxidant capacity and upregulates steroidogenic enzyme expression in Leydig cells of aged roosters by activating the MAPK-Nrf2 pathway (Ma et al., 2024). Oxidative stress directly inhibits testosterone synthesis by damaging Leydig cell mitochondrial function and downregulating steroidogenic enzyme expression, and the decline of antioxidant components in aged Leydig cells is closely associated with the deterioration of testosterone synthesis (Luo et al., 2006). Therefore, integrating the coordinated pattern of Nrf2 pathway gene upregulation, elevated HSD17B3 expression, and increased serum testosterone levels observed in this study, together with the established associations between the Nrf2 pathway and Leydig cell steroidogenesis in the literature, we speculate that DNP may be associated with Nrf2 pathway activation, which may in turn be related to improvements in the redox status of Leydig cells and the upregulation of HSD17B3 expression.

The systemic improvement in serum and testicular antioxidant indices may be an important associated factor for the maintenance of reproductive performance in the DNP group. In serum, CAT activity of the DNP group was elevated by 40 d, and by 60 d, both CAT and GSH-Px activities were higher than those of the control group, while MDA levels were reduced, indicating that the DNP group exhibited systemic antioxidant improvement from the mid-period onward. The response of antioxidant enzymes to supplementation is time-dependent, with different enzyme classes exhibiting varying response rates (Golestani et al., 2006). On 0 d, the T-SOD activity of the control group was higher than that of the DNP group, and this difference disappeared by 60 d, suggesting that T-SOD activity in the DNP group gradually recovered to a level comparable to that of the control group. This delayed recovery may be related to the fact that SOD, as a first-line enzyme in ROS scavenging, has more complex regulatory mechanisms and is more sensitive to changes in redox status (Feng et al., 2026). In testicular tissue, the activities of CAT, GSH-Px, and T-SOD, as well as T-AOC levels, were all elevated in the DNP group, while MDA levels were reduced; furthermore, testicular MDA was negatively correlated with sperm motility and concentration, suggesting that the reduction in lipid peroxidation levels may contribute to maintaining the structural integrity of spermatogenic cell membranes. However, HO-1 mRNA expression did not differ between the two groups, which may reflect selective regulation of Nrf2 downstream target genes; under aging conditions, HO-1 induction may require stronger oxidative stress stimuli or the involvement of different transcriptional coactivators (Ren et al., 2026).

To further investigate the upstream regulatory mechanisms by which DNP enhances testicular antioxidant capacity, this study examined the mRNA expression of Nrf2 pathway-related genes. The expression of Nrf2 and its downstream target genes NQO-1, GSTA3, SOD1, and CAT were all upregulated in the DNP group, suggesting an association between Nrf2 pathway activation and the enhancement of the testicular antioxidant defense network in the DNP group. Nrf2 is the core transcription factor in the cellular response to oxidative stress; under physiological conditions, it is bound by Keap1 and degraded via ubiquitination. Upon stimulation by oxidative stress or induction by antioxidants, Nrf2 dissociates from Keap1, translocates to the nucleus, and binds to antioxidant response elements (**ARE**) to initiate the transcription of phase II detoxification enzymes and antioxidant proteins (Kobayashi et al., 2004; Ma, 2013). GSTA3, as a target gene of Nrf2, promotes the clearance of lipid peroxidation products and electrophilic substances through glutathione conjugation reactions; CAT is a first-line enzymatic defense system for intracellular ROS scavenging (Sies, 2015). Previous studies have demonstrated that Dendrobium polysaccharides can alleviate cyclophosphamide-induced oxidative damage to the male reproductive system by activating the Nrf2/ARE pathway (Mu et al., 2023), and the results of the present study are consistent with these findings.

Untargeted metabolomics analysis provided clues regarding the potential association between DNP and changes in the testicular metabolic microenvironment. Differential metabolites were primarily enriched in amino acid biosynthesis and cysteine and methionine metabolism pathways, suggesting a potential association between alterations in amino acid metabolic pathways and improvements in testicular antioxidant capacity. Cysteine is the rate-limiting precursor for glutathione (**GSH**) synthesis, and the enrichment of its metabolic pathway suggests that increased endogenous GSH supply may be synergistically associated with the antioxidant effects following Nrf2 pathway activation (Lu, 2013). Correlation analysis further revealed associations among metabolites, antioxidant parameters, and semen quality. Acetylcarnitine, creatine, and DL-proline were positively correlated with most antioxidant parameters and negatively correlated with MDA, whereas DL-cysteine, fructose, and kojic acid exhibited the opposite pattern. Acetylcarnitine transports long-chain fatty acids into mitochondria for β-oxidation to generate energy while simultaneously possessing antioxidant properties (Neuman et al., 2002); creatine buffers ATP fluctuations through the phosphocreatine system, maintaining the energy homeostasis required for sperm motility (Kaldis et al., 1996). The association patterns between these metabolites and antioxidant parameters and semen quality suggest that they may participate in the process by which DNP improves the testicular microenvironment; however, causal relationships require further verification. The negative correlation between DL-cysteine and sperm motility appears to contradict the antioxidant role of cysteine as a GSH precursor. However, the redox behavior of cysteine is concentration-dependent. At physiological concentrations, cysteine functions as the rate-limiting precursor for GSH synthesis, exerting antioxidant effects (Lu, 2013); when concentrations exceed the utilization threshold, excess cysteine undergoes copper- or iron-catalyzed autoxidation, generating large quantities of H2O2 and shifting to a pro-oxidant effect (Nath and Salahudeen, 1993). Avian sperm membranes are rich in polyunsaturated fatty acids and are highly susceptible to H2O2-mediated lipid peroxidation (Surai et al., 1998); excessive accumulation of free cysteine in the testis may be related to this susceptibility and is potentially associated with sperm membrane damage and reduced motility. The association between DNP and Nrf2 pathway activation may be further linked to the metabolic flux direction of cysteine toward GSH synthesis, a speculation that awaits confirmation through functional validation experiments.

## Conclusion

In summary, dietary supplementation with Dendrobium nobile polysaccharide is associated with the upregulation of Nrf2 pathway-related gene expression and improvements in antioxidant capacity, and is associated with elevated testosterone levels, which may contribute to delaying the decline of reproductive function in aged breeding roosters. Metabolomics analysis suggests a potential association between alterations in amino acid metabolic pathways and the antioxidant protective effects of DNP, and the specific regulatory mechanisms warrant further investigation. This study provides preliminary experimental evidence supporting the use of DNP as a natural functional feed additive for extending the reproductive lifespan of breeding roosters.

## Funding

This work was supported by the Key Project of the Science and Technology Support Program of Guizhou Provincial 10.13039/100015811Department of Science and Technology (Qiankehe Support [2022] Key 034, to H.L.) and the Guizhou Provincial Department of 10.13039/501100000094Agriculture and Rural Affairs (Qiannong [2026] No. 01, to H.L.).

## Ethics statement

Ethical approval for the animal protocol was granted by the Animal Experiment Management and Use Committee of Guizhou University (Guiyang, China; Guizhou Science and Technology Support Project [2022] Key 034; Guizhou Agriculture [2026] No. 01). All animal procedures were conducted in full compliance with the Regulations on the Management and Use of Laboratory Animals established by the Ministry of Agriculture and Rural Affairs of the People’s Republic of China.

## Credit authorship contribution statement

**Jilong Zhang:** Writing – original draft, review, data curation, conceptualization. **Depeng Zhao:** Writing – original draft, review, data curation, conceptualization. **Yushi Shi:** Review, data curation, conceptualization. **Xia Long:** Review, data curation. **Qisong Tan:** Review, data curation. **Jinlin Yang:** Data curation. **Gang Feng:** Data curation. **Beida Zhou:** Data curation. Hui Li*: Writing – review & editing, funding acquisition, conceptualization.

## Disclosures

The authors declare that they have no known competing financial interests or personal relationships that could have appeared to influence the work reported in this paper.

## Data Availability

The data that support the findings of this study are available from the corresponding author upon reasonable request.

## Footnotes

Supplementary material associated with this article can be found, in the online version, at doi:10.1016/j.psj.2026.107602.
