# Supplementary material for: Effects of dendrobium nobile polysaccharide on reproductive performance, hormone levels, antioxidant capacity, and testicular metabolism of aged roosters
Source: Poult Sci. 2026 Aug 11;105(12):107602. doi: 10.1016/j.psj.2026.107602 (PMC13594879; doi:10.1016/j.psj.2026.107602)
Supplement: Supplementary file 1 [file mmc1.docx]

Supplementary Table 1. Primer sequences for RT-qPCR

| **Gene** | **Primer sequences (5' to 3')** | **Product size(bp)** | **GenBank** |
| --- | --- | --- | --- |
| ***NRF2*** | F: CTGTCTTCAGCCTTTTTGGACA  R: AAAAACTTCACGCCTTGCCC | 22  20 | NM_001396902.1 |
| ***HO-1*** | F: CACACAACGCTGAAAGCATGTC  R: AACTCGTGGAGGGACACCT | 22  19 | NM_205344.2 |
| ***NQO-1*** | F: AACCTCTTTCAACCACGCCA  R: TTCTTGAGGGGTCCGGTGAT | 20  20 | NM_001277619.2 |
| ***GSTA3*** | F: CGTCGTCCAACAGCAGATA  R: CCGTGGTCCTTCAAAACCTTC | 20  21 | NM_001001777.2 |
| ***SOD1*** | F: TGACCTCGGCAATGTGACTG  R: CACTTTTTGCATGGACCACCA | 20  21 | NM_205064.2 |
| ***CAT*** | F: GCTGAAGCTGGGAAAAAGGATG  R: TCCTGCAGTTGTATGGACGC | 22  20 | NM_001031215.2 |
| ***HSD17b3*** | F: GAGAATGGGCAGTGGTCACA  R: TATTTAGCCCGCGTTTTGCC | 20  20 | XM_425046.8 |
| ***β-actin*** | F: GAGAAATTGTGCGTGACATCA  R: CCTGAACCTCTCATTGCCA | 21  19 | NM_205518.2 |
